# Rare variants in cardiomyopathy genes predispose to cardiac injury in severe COVID-19 patients of African or Hispanic ancestry

**DOI:** 10.1007/s00109-024-02510-z

**Published:** 2024-12-27

**Authors:** Hui-Qi Qu, Matthew S. Delfiner, Chethan Gangireddy, Anjali Vaidya, Kenny Nguyen, Isaac R. Whitman, JuFang Wang, Jianliang Song, Michael R. Bristow, Charles F. McTiernan, Glenn S. Gerhard, Hakon Hakonarson, Arthur M. Feldman

**Affiliations:** 1 The Center for Applied Genomics, Children’s Hospital of Philadelphia, Philadelphia, PA USA; 2 Department of Medicine, Division of Cardiology, Lewis Katz School of Medicine at Temple University, Philadelphia, PA USA; 3 Division of Cardiology, University of Colorado Anschutz Medical Campus, Aurora, CO USA; 4 Division of Cardiology, Heart and Vascular Institute, University of Pittsburgh, Pittsburgh, PA USA; 5 Department of Human Genetics and Molecular Biochemistry, Lewis Katz School of Medicine at Temple University, Philadelphia, PA USA; 6 Division of Human Genetics, Children’s Hospital of Philadelphia, Philadelphia, PA USA; 7 Department of Pediatrics, Perelman School of Medicine, University of Pennsylvania, Philadelphia, PA USA

**Keywords:** COVID-19, SARS-CoV-2, Myocardial injury, Cardiomyopathy, African

## Abstract

**Abstract:**

In one of the earliest reports from China during COVID-19, it was noted that over 20% of patients hospitalized with the disease had significant elevations of troponin, a marker of myocardial tissue damage, that put them at a higher risk. In a hypothesis-independent whole exome sequencing (WES) study in hospitalized COVID-19 patients of diverse ancestry, we observed putative enrichment in pathogenic variants in genes known to be involved in the pathogenesis of cardiomyopathy. This observation led us to hypothesize that the observed high morbidity and mortality in these patients might be due to the presence of rare genetic factors that had previously been silent but became relevant as a consequence of the severe stress inflicted by an infection with SARS-CoV-2. To test this hypothesis, we analyzed our WES data generated from a cohort of 325 patients sequentially admitted for COVID-19 infection. In this predominantly minority population (53.9% African ancestry and 37.9% Hispanic/Latin ancestry), our initial analysis screen identified 263 variants that were identified as highly deleterious (HD) from a total of 26,661 variants of interest that represented 215 genes. Of those, we identified 46 genes (in 58 patients) harboring rare HD coding variants that were previously implicated in dilated cardiomyopathy and were considered as disease initiators for the severe COVID-19 in this study. These findings offer valuable insights into the molecular mechanisms and genetic susceptibility to heart injury in severe COVID-19.

**Key messages:**

- COVID-19 may cause cardiac damage in some affected patients without a plausible biological explanation.
- Our study reveals an enrichment of highly deleterious variants linked to cardiomyopathy in severe COVID-19 patients.
- Genetic profiling unveils the molecular basis of severe COVID-19-related heart injury, potentially aiding in patient stratification.

**Supplementary Information:**

The online version contains supplementary material available at 10.1007/s00109-024-02510-z.

## Introduction

The severe acute respiratory syndrome virus-2 (SARS-CoV-2) has infected over 62 million people in the USA and the resulting disease, COVID-19, has contributed to the deaths of millions worldwide [1]. Most COVID-19 patients have had relatively minor symptoms, but typical of other respiratory viruses, a subgroup presented with acute respiratory distress syndrome (ARDS) requiring hospitalization and an admission to an intensive care unit [2]. Consistent with previous studies with other respiratory viruses, mortality was worse in patients who were older and had a history of prior pulmonary disease [3]. However, early reports from China noted that 19.7% of hospitalized COVID-19 patients also had increased levels of high sensitivity troponin I (hsTnI), suggesting that these patients had sustained myocardial damage [4, 5]. Although many of the patients had typical risk factors for cardiovascular disease, the group also included young patients without known risk factors. Increased levels of C-reactive protein (CRP), consistent with systemic inflammation, and high levels of N-terminal pro-B-type natriuretic peptide (NT-proBNP), suggesting myocardial congestion, were also present. As COVID-19 spread to the USA, a study of 2736 hospitalized COVID-19 patients in New York found that 36% had elevated cardiac troponin I (cTnI) concentrations and that even a small elevation was associated with an increased risk of death [6]. However, there was no plausible biological explanation for why only a small number of people developed cardiac damage after an infection with SARS-CoV-2 [7].

Recognizing the complexity of the cardiac inflammasome and the relatively modest number of COVID-19 patients who were hospitalized with cardiac damage, we hypothesized that the group of COVID-19 patients who develop cardiac damage had a pre-existing risk that made them susceptible to the cardiac stress of a severe viral infection. We also based our hypothesis on our knowledge of another enigmatic disease: post-partum cardiomyopathy (PPCM). In 1990, we reported for the first time that 14 of 18 women with PPCM had myocarditis on an initial endomyocardial biopsy (EMB) [8, 9]; however, most had no serious abnormalities on repeat biopsy six weeks later. Those patients whose repeat biopsies were normal had the eventual restoration of normal LV function whereas those who failed to clear their inflammation had persistent HF symptoms requiring transplantation. In a similar patient population to that of PPCM, Ware et al. found that 15% of the women had truncating variants of putative functional relevance, with the most frequent variants being found in the well-established cardiomyopathy gene, *Titin* (*TNN*) [10], a finding confirmed in a subsequent study [11].

As the results of these prior studies would suggest that similar underlying genetic variances might be a cause for the myocardial injury in a subset of patients with COVID-19, we elected to search for disease causing variants in our cohort. By contrast with earlier studies in myocarditis that focused on an established group of genes, the unique features of infections with COVID-19 led us to look more broadly for potentially causative variants. We therefore used WES to investigate the presence of genetic variants in inpatients with COVID-19 who were admitted to a large urban hospital that cares for a largely minority population. Not only is this among the first studies to systematically decipher the role of genetics in severe COVID-19 but it is also the first genomics study to focus a genetic analysis on minority populations that have been severely under-represented in genetic analyses to this date.

## Materials and methods

### Patients

Patients admitted consecutively between March 15, 2020 and July 26, 2020 were included in this analysis. The COVID-19 Cardiac Outcomes study (COVID CARDOS) was a retrospective registry of hospitalized COVID-19 patients that had an a priori goal of collecting and curating hospital records from 500 subjects. All hospitalized patients were 18 years of age or older and diagnosed with COVID-19 by polymerase chain reaction (PCR). Clinical datapoints included demographic information, medical history, medications on admission, laboratory values at admission, inpatient treatments including supplemental oxygen, patient disposition, and laboratory values throughout the hospitalization. Subjects were noted to have myocardial injury if their initial cTnI level was greater than the 99th percentile of the normal value. Patients were excluded from the registry if key demographic or clinical datapoints were missing from the medical record. All clinical data were obtained from the electronic medical record, anonymized by an independent broker, and entered into a secure database with REDCap. This study was performed in line with the principles of the Declaration of Helsinki. The Temple University Institutional Review Board (IRB) approved the protocol. All the patients were recruited before the availability of COVID-19 vaccines.

### DNA collection and processing

Blood was collected as part of a separate IRB approved biobank from COVID-19 inpatients. In brief, EDTA collection tubes from which a small sample had been removed for automated blood analyses were collected daily by research personnel from the CLIA certified genomics laboratory. The patient identifier on each tube was removed and a replacement identifier affixed. The prior identifier was entered into the secure database, which was then transmitted to the independent broker, who then entered the information into a secure REDCap database. In this way, only the independent broker was able to link a blood sample to clinical data and neither representatives from the clinical registry nor the biobank had the ability to cross from one data set to the other. The collection tubes were stored at – 70 °C in the Human Genetics Biobank until such time as they were sequenced. DNA was isolated using the QIAGEN QIAamp Blood Mini protocol according to the manufacturer’s instructions. After thawing, blood was incubated with QIAGEN Protease or proteinase K at 56 °C for 10 min, 96–100% ethanol was added for loading on to a QIAamp Mini spin column followed by centrifugation at 6000 × g for 1 min. One wash buffer was applied followed by centrifugation at 6000 × g for 1 min with a second wash buffer at 20,000 × g for 3 min followed by a second spin for 1 min. DNA was eluted from the column using QIAGEN elution buffer after incubation at room temperature (15–25 °C) for 5 min and centrifugation at 6000 × g for 1 min. Eluted DNA was stored frozen at – 20 °C. A third IRB approval allowed for the independent broker to provide the requisite data for the analyses performed as part of this study by coupling genetic data with clinical outcome data from the same patient with all personal identifiers removed.

### Exome sequencing

Exome sequencing was done by the Helix Exome + ® v2 Assay (San Mateo, CA). Illumina NovaSeq was used for paired end sequencing (San Diego, CA). Data were processed using Sentieon DNASeq Variant Calling Workflow (https://www.sentieon.com/) and aligned to GRCh38. The WES callings were annotated using the ANNOVAR software [12]. Genetic variants which have minor allele frequency (MAF) less than 0.001 in the African ancestry group (the largest racial group), based on the Exome Aggregation Consortium (ExAC) database, were identified [13]. Functional candidate variants were selected by the prediction results with at least 1 of a number of genetic variant prediction software, i.e., SIFT_pred = "D" or Polyphen2_HDIV_pred = "D" or Polyphen2_HDIV_pred = "P" or Polyphen2_HVAR_pred = "D" or Polyphen2_HVAR_pred = "P" or LRT_pred = "D" or MutationTaster_pred = "A" or MutationTaster_pred = "D" or MutationAssessor_pred = "H" or MutationAssessor_pred = "M" or FATHMM_pred = "D" or PROVEAN_pred = "D" or MetaSVM_pred = "D" or MetaLR_pred = "D", based on the annotation with the ANNOVAR software, as we have previously described [14, 15].

### Data analysis

To determine the classification of pathogenic (P) or likely-pathogenic (LP) variants the rare coding variants in the sample were annotated with the InterVar software [16] for automating functional classification of sequence variants by the ACMG criteria, as well as the most recent data release of the Human Gene Mutation Database (HGMD_Pro_2022.1_hg38) [17]. Pathogenic (P) or likely pathogenic (LP) variants were also identified using ClinVar annotations, as described in detail, previously [18, 19]. Coding variants classified as pathogenic (P) or likely pathogenic (LP) were accepted only when identified by both InterVar/HGMD and ClinVar.

To identify the burden of rare coding variants, supplementary to the approach for Highly Deleterious (HD) variants, burden of functional rare coding variants was tested in the cases against the frequencies in the ExAC African ancestry population, and the frequencies in the ExAC Non-Finish European (NFE) population, respectively. Functional rare coding variants identified based on the ANNOVAR software were counted in the cases with the Test Rare vAriants with Public Data (TRAPD) software [20], and compared with the ExAC African ancestry and NFE controls under the dominant inheritance model by one tailed Fisher exact test. We have optimized the TRAPD algorithm with normalized genome coverage to capture causal variants with effects in the same directions [14, 15]. We corrected significance level for multiple testing by Bonferroni correction. The genome-wide significance of the gene-based burden test was defined as α = 0.05/21,306 = 2.35E-06, assuming 21,306 protein-coding genes in the human genome [21]. Due to the racial heterogeneity of the research subjects, burden of rare coding variants in cases were tested against both African ancestry and NFE populations. Significance for a gene was required for both tests against African ancestry and NFE populations. Over-representation analysis (ORA) was done using the WebGestalt (WEB-based Gene SeT AnaLysis Toolkit) web tool by the human phenotype ontology (HPO) approach [22]. Statistics analysis for the multinomial logistic regression was done using the IBM SPSS Statistics Version 23 software.

## Results

### Study subjects

Of the 500 patients included in a cardiac clinical registry established to study COVID-19 patients, 325 had biological samples available for genetic analysis. Ages ranged from 18 to 90 years old, and there were 175 (53.9%) patients identified as African American (AA), 23 (7.1%) as White, 2 (0.6%) as Asian, 123 (37.9%) as Hispanic/Latin, and 2 (0.6%) were undetermined. Of the total, 139 (42.8%) were Female. In the cohort of 325 patients evaluated in this study, 175 (53.9%) were of African ancestry of whom 74 out of 164 measured (45.1%) had positive plasma troponin at admission; 89 of 168 (53.0%) had positive plasma troponin at some point in their hospitalization (i.e., 4 additional patients had troponin levels measured during the hospital stay but not on admission). Among non-African American (non-AA) patients, 48 out of 140 (34.3%) measured had positive plasma troponin at admission, while 61 out of 143 (42.7%) had positive plasma troponin at some point during their hospitalization.

As shown in Table 1, the baseline characteristics of the AA population were comparable to those of the Hispanic ancestry group except for a higher percentage of patients of African ancestry had hypertension and chronic kidney disease when compared with the non-AA group. Similarly, clinical outcomes were not different between the two groups. Interestingly, 40.8% of the COVID-19 patient population had myocardial injury as evidenced by an elevated troponin at admission. That troponin was a useful tool for identifying patients with myocardial damage as evidenced by the finding that twice (27.3%) the number of individuals who had serum troponin detected at the time of admission died in the hospital compared to 12.4% deaths amongst those who did not have troponin leaks at the time of admission (*P* = 0.021 by binary logistic regression, corrected by age and BMI).

**Table 1:** Clinical characteristics, laboratory values and clinical outcomes

	African American (*n* = 197)	Non-African American (*n* = 173)	*p* value
a. Baseline characteristics and presenting laboratory values			
Age (years)	61.5 (14.3)	61.0 (15.1)	0.75
Female gender	94 (48)	67 (39)	0.90
Hypertension	164 (84)	108 (62)	< 0.001
Chronic kidney disease	52 (26)	28 (38)	0.02
Coronary artery disease	28 (14)	34 (20)	0.16
Diabetes mellitus	89 (45)	77 (45)	0.56
History of stroke	27 (14)	26 (15)	
Heart failure with reduced Ejection fraction	21 (11)	15 (9)	0.53
Heart failure with preserved ejection fraction	22 (11)	22 (13)	0.61
Body mass index (kg/m^2^)	32.1 (9.2)	31.1 (7.4)	0.27
Troponin (ng/mL)*	0.69 (2.3)	1.6 (15)	0.37
Elevated Troponin*	82 (42)	57 (33)	0.08
B-type natriuretic peptide (pg/mL)*	244 (636)	336 (707)	0.24
Elevated B-type natriuretic peptide*	55 (27)	54 (31)	0.88
C reactive protein (mg/dL)^#^	13.5 (43.2)	13.9 (28.2)	0.92
b. Clinical outcomes			
Lactic acid (mmol/L)^#^	1.8 (1.5)	1.9 (1.3)	0.76
Inpatient mortality	38 (19)	28 (16)	0.450
Intensive care	72 (37)	64 (37)	0.900
Intubation	40 (20)	25 (14)	0.146
Supplemental oxygen	143 (73)	130 (75)	0.398
Non-invasive positive pressure ventilation	35 (18)	41 (24)	0.152
High flow oxygen	62 (31)	61 (35)	0.420
c. WES	n = 175	n = 150	

Continuous variables noted as mean (standard deviation). Categorical variables noted as number (percent). *Elevated troponin determined by values > 0.012 ng/mL. Elevated B-type natriuretic peptide (BNP) determine by values > 100 pg/mL. Troponin and BNP are peak levels during hospital stay.^#^ C reactive protein and lactic acid are levels at admission

To conduct meaningful genetic analyses, ensuring homogeneity within cohorts is essential for the accuracy and reliability of our findings. For this reason, we stratified participants into two groups: AA and non-AA. This distinction recognizes the ancestral diversity of human populations, with African populations representing the root lineage for all modern humans based on evolutionary and genetic evidence. Dividing the cohort in this manner allows us to investigate potential genetic and prognostic variations between these groups, which may influence health outcomes in the context of COVID-19. By integrating evolutionary biology with socioeconomic considerations related to race, our approach aims to illuminate the complex interplay between genetic predispositions and environmental or systemic factors contributing to health disparities. For example, hypertension, a major risk factor for severe COVID-19, is more prevalent in African American populations (Table 1). Additionally, genetic analyses revealed differences in the frequencies of HD variants in cardiomyopathy genes in AA and non-AA participants. This genetic heterogeneity further highlights the importance of stratified analyses by population groups to ensure robust and meaningful insights.

### Sequencing

The sequencing was done in both the AA and non-AA cohorts. In this cohort, we identified 26,661 deleterious rare coding variants in 10,522 genes with allele frequencies in the African ancestry population of < 0.1%. Allele frequencies of functional variants can vary significantly across human populations due to evolutionary pressures and genetic drift [23]. Given the large representation of African American participants in this study, we opted to use allele frequencies from the African ancestry population to filter out common variants that are less likely to have a significant functional impact. Frequencies derived from other populations, such as European or Asian populations, may be influenced by positive or purifying selection related to their significant effects, which could lead to the misclassification of certain variants as rare or common. Variants were considered HD if, upon data aggregation, they were identified as deleterious by two of the following: ClinVar, HGMD classification (HGMD-Pro-2022.1-hg38), or InterVar software. (Supplementary Data 1).

### Variants in cardiomyopathy genes

**Fig. 1:**
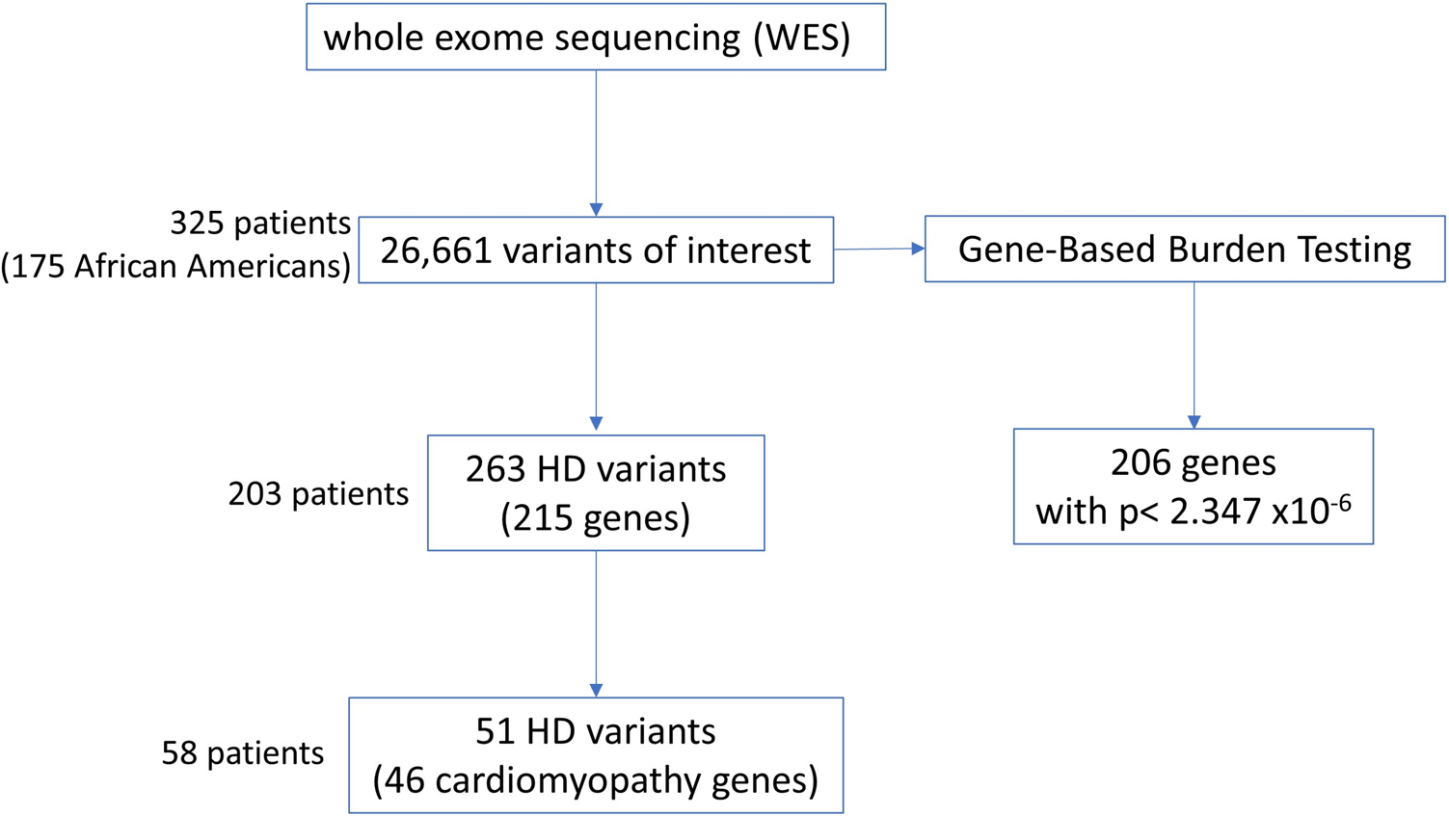
Algorithm of genetic variant analysis in this study. By whole exome sequencing (WES), 26,661 variants of interest (VOI) were identified. Among these VOIs, 263 variants in 215 genes were identified as highly deleterious (HD). Over-representation analysis by Human Phenotype Ontology (HPO) identified 51 HD variants from 46 genes in the HPO gene set Cardiomyopathy (HP:0001638). In parallel, mutation burden analysis of the 26,661 coding variants identified 206 genes with significant *p* values (*p* < 2.347 × 10.^−6^)

From the 26,661 variants of interest, we identified 263 variants from 215 genes as HD (Fig. 1). The 263 HD variants included 200 nonsynonymous variants, 46 stop/gain variants, and 17 splicing variants (Fig. 1). HD variants were seen in 88 of 175 patients of African ancestry (50.3%: Supplementary Data 2) and 115 of the 150 non-African ancestry patients (76.6%). (Supplementary Data 2).

**Fig. 2:**
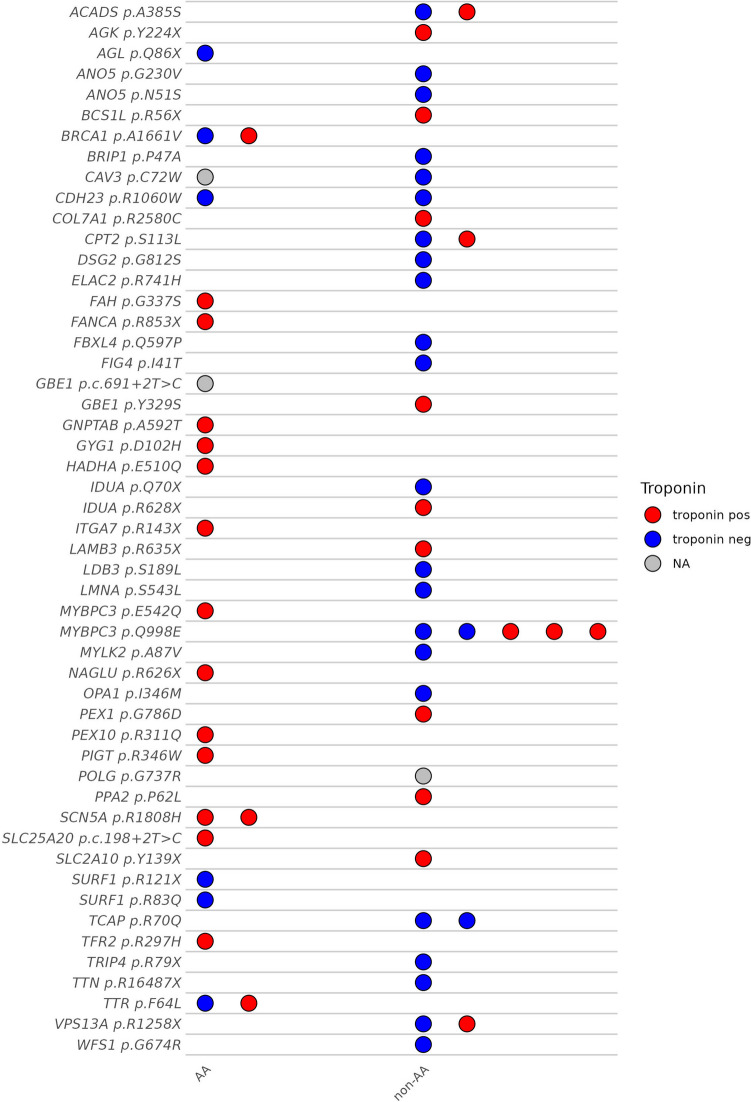
Distribution of HD variants in each group. Each circle represents an individual with a mutation, with the corresponding troponin status indicated by color

Using the WebGestalt (WEB-based Gene SeT AnaLysis Toolkit) web tool, we performed over-representation analysis (ORA) using the Human Phenotype Ontology (HPO) pre-specified phenotype terms [24], for the 215 genes identified as HD variants in the COVID-19 patients. Among10 gene sets found to be enriched for statistical significance (Supplementary Table 1), the most highly enriched gene set was cardiomyopathy (HP:0001638) which had a false discovery rate (FDR) of 7.08 × 10^−05^. Included in this gene set were 46 genes that potentially play roles in cardiomyocytes, all of which harbor HD variants as determined by the three programs used in this study (Fig. 2, Supplementary Table 2). The HD variants in these 46 genes were detected in 58 patients. Twenty-two of these patients were of African ancestry (37.9%) and were positive for at least one of these variants. Interestingly, in the patients harboring HD variants in these cardiomyopathy genes, we observed a significantly higher incidence of elevated troponin levels during their hospitalization [16 out of 21 patients with HD variants (76.2%) vs. 73 out of 147 patients without HD variants (49.7%) in African Americans, *p* = 0.020]. Covariate analysis showed that age was a risk factor for a positive troponin (*p* = 0.014), but BMI (*p* = 0.597) or grades of obesity (*p* = 0.487) were not risk factors.

### Mutation burden analysis of rare coding variants

In parallel with the analysis we describe above, we performed mutation burden analysis of all of the 26,661 rare functional coding variants, to prioritize candidate genes. We identified 206 genes with significant p values (*p* < 2.35 × 10^−06^) when compared to both African ancestry and the ExAC Non-Finish European (NFE) reference population (Supplementary Data 3). Among these 206 genes, 16 genes overlapped with the list of 215 genes with HD variants. In addition, 46 cardiomyopathy genes identified as harboring HD variants showed varied significance of burden with coding variants (Table 2). Two mutations, i.e., caveolin 3 (*CAV3*) C72W and cadherin related 23 (*CDH23*) R1060W, were identified in patients of African and non-African ancestry. Furthermore, 2 of the 46 cardiomyopathy genes, *CDH23* and myosin binding protein C (*MYBPC3*), were significantly enriched for rare functional coding variants, as revealed by mutation burden analysis (Supplementary Data 3). Our further experimental research on these two genes in heart failure is described in the supplementary methods and results.

**Table 2:** Cardiomyopathy genes identified of HD variants and burden with coding variants

Gene Symbol	Variant	Count_Afr	Count_NonAfr	subtotal	*Heterogeneity P* (2 tailed Fisher's)	ExAC_AFR	ExAC_Freq (all)
*ACADS*	A385S	0	2	2	0.212	0	4.33E-05
*AGK*	Y224X	0	1	1	0.462	0	2.49E-05
*AGL*	Q86X	1	0	1	1.000	3.00E-04	2.48E-05
*ANO5*	N51S	0	1	1	0.462	7.00E-04	1.90E-03
	G230V	0	1	1	0.462	2.00E-04	1.10E-03
*BCS1L*	R56X	0	1	1	0.462	9.61E-05	2.00E-04
*BRCA1*	A1661V	2	0	2	0.501	4.00E-04	3.32E-05
*BRIP1*	P47A	0	1	1	0.462	0	2.00E-04
*CAV3*	C72W	1	1	2	1.000	6.00E-04	1.10E-03
*CDH23*	R1060W	1	1	2	1.000	2.00E-04	4.00E-04
*COL7A1*	R2580C	0	1	1	0.462	0	3.96E-05
*CPT2*	S113L	0	2	2	0.212	2.00E-04	1.30E-03
*DSG2*	G812S	0	1	1	0.462	0	1.66E-05
*ELAC2*	R741H	0	1	1	0.462	4.00E-04	5.00E-04
*FAH*	G337S	1	0	1	1.000	3.00E-04	1.00E-04
*FANCA*	R853X	1	0	1	1.000	9.69E-05	8.26E-06
*FBXL4*	Q597P	0	1	1	0.462	0	1.65E-05
*FIG4*	I41T	0	1	1	0.462	9.91E-05	1.00E-03
*GBE1*	Y329S	0	1	1	0.462	0	3.00E-04
	c.691 + 2 T > C	1	0	1	1.000	0	1.10E-03
*GNPTAB*	A592T	1	0	1	1.000	0	1.00E-04
*GYG1*	D102H	1	0	1	1.000	2.00E-04	9.00E-04
*HADHA*	E510Q	1	0	1	1.000	4.00E-04	1.20E-03
*IDUA*	R628X	0	1	1	0.462	0	3.35E-05
	Q70X	0	1	1	0.462	1.00E-04	7.00E-04
*ITGA7*	R143X	1	0	1	1.000	2.00E-04	4.12E-05
*LAMB3*	R635X	0	1	1	0.462	2.00E-04	8.00E-04
*LDB3*	S189L	0	1	1	0.462	0	6.00E-04
*LMNA*	S543L	0	1	1	0.462	0	1.00E-04
*MYBPC3*	Q998E	0	5	5	0.020*	4.00E-04	5.20E-03
	E542Q	1	0	1	1.000	1.00E-04	2.49E-05
*MYLK2*	A87V	0	1	1	0.462	3.00E-04	1.00E-04
*NAGLU*	R626X	1	0	1	1.000	2.00E-04	2.74E-05
*OPA1*	I346M	0	1	1	0.462	4.00E-04	6.00E-04
*PEX1*	G786D	0	1	1	0.462	9.71E-05	3.00E-04
*PEX10*	R311Q	1	0	1	1.000	5.00E-04	3.77E-05
*PIGT*	R346W	1	0	1	1.000	0	1.65E-05
*POLG*	G737R	0	1	1	0.462	3.00E-04	7.00E-04
*PPA2*	P62L	0	1	1	0.462	9.89E-05	2.00E-04
*SCN5A*	R1808H	2	0	2	0.501	1.00E-04	3.31E-05
*SLC25A20*	c.198 + 2 T > C	1	0	1	1.000	6.00E-04	4.94E-05
*SLC2A10*	Y139X	0	1	1	0.462	0	8.24E-06
*SURF1*	R83Q	1	0	1	1.000	0	1.80E-05
	R121X	1	0	1	1.000	9.63E-05	2.00E-04
*TCAP*	R70Q	0	2	2	0.212	0	2.09E-05
*TFR2*	R297H	1	0	1	1.000	9.83E-05	7.00E-04
*TRIP4*	R79X	0	1	1	0.462	0	2.47E-05
*TTN*	R16487X	0	1	1	0.462	0	8.34E-06
*TTR*	F64L	2	0	2	0.501	6.00E-04	5.77E-05
*VPS13A*	R1258X	0	2	2	0.212	0	8.24E-06
*WFS1*	G674R	0	1	1	0.462	0	3.00E-04

^*^Among the 51 variants from the 46 genes, 28 are currently classified as P/LP, 16 with conflicting interpretations of pathogenicity (CIP), 6 as uncertain significance (VUS), and 1 as Benign/Likely benign. All the 16 CIP variants were previously classified as P/LP by ClinVar according to ANNOVAR, while 4 were predicted as LP by InterVar, and the left were classified as DM by HGMD. All the 6 VUS were previously classified as P/LP by ClinVar according to ANNOVAR, while 1 was predicted as LP by InterVar, and the left were classified as DM by HGMD. The 1 B/LB variant was previously classified as P/LP by ClinVar according to ANNOVAR, and classified as DM by HGMD. In particular, the controversial B/LB variant in *MYBPC3* was only seen in non-African individuals, and was not seen in African Americans that we demonstrated of genetic correlation with elevated troponin. Burden test of rare VOIs in *MYBPC3* has genome-wide significance as well. More information is shown in Supplementary Data 2. The 46 genes are included in all these HPO genesets: Abnormality of cardiovascular system morphology (HP:0030680); Abnormal heart morphology (HP:0001627); Abnormal myocardium morphology (HP:0001637); Cardiomyopathy (HP:0001638); Abnormality of the cardiovascular system (HP:0001626)

## Discussion

This analysis of severe COVID-19 patients represents a groundbreaking discovery, as it unveils a genetic predisposition that is notably enriched for cardiomyopathy-associated genes, all identified through hypothesis-independent analysis. Building upon this compelling observation of heightened cardiomyopathy gene enrichment, we conducted a targeted investigation into “well-defined” cardiomyopathy genes using a hypothesis-driven approach. In this regard, we hypothesized that patients who developed myocardial injury during an infection with the SARS-CoV-2 virus carried underlying variants in cardiac genes that limited the heart’s ability to support normal cardiac output during the stress of a severe viral infection. A corollary to this underlying hypothesis was that the abundance of genetic variation in the human genome accounts for a wide range of responses to physiologic stress—in this case to the stress of a severe viral infection [25]. While the number of HD variants in this patient cohort is high, it is important to note that these hospitalized for COVID-19 patients were all very sick and there were no COVID-19 specific treatments at the time of the study conduct. On a single day at the mid-point of the data collection there were over 500 new cases in the Philadelphia County averaging about 40 deaths per day, and Temple University carried the highest census as all elective procedures had been discontinued and a 100 bed unit within the hospital was established only to treat COVID-19. Therefore, it would not be unexpected to see a significant number of maladaptive genetic variants in a population enriched for SARS-CoV-2 infected patients, including severe pulmonary and cardiac disease.

Among the characteristic features of patients who were hospitalized for COVID-19 was that they demonstrated an elevation of cTnI. The choice of troponin as a biomarker was supported by a recent study performed at four hospitals in New York that enrolled 2736 COVID-19 patients and used measures of cTnI as a marker of myocardial injury [6]. They found that small elevations of troponin representing limited myocardial injury were associated with death while greater amounts of troponin were associated with a higher risk of death. Their results were consistent with earlier studies [26] as well as with the present study, in which we found that an increase in cTnI levels was associated with a worse outcome.

The present study is the first to identify a spectrum of rare HD variants in the landscape of COVID-19 myocardial injury. Among these, 10 genes, *CAV3*, *DSG2*, *LDB3*, *LMNA*, *MYBPC3*, *MYLK2*, *SCN5A*, *TCAP*, *TTN*, and *TTR*, have been defined as the associated genes of cardiomyopathy by the 2023 European Society of Cardiology (ESC) Guidelines [27]. The remaining 36 genes, though not explicitly recognized by the ESC Guidelines, are implicated in diverse mechanisms that maintain cardiac structure and function. These mechanisms include contributions to the stability of sarcomeric and extracellular matrix proteins, which preserve the structural integrity of cardiomyocytes and prevent damage caused by mechanical stress [28–30]. Dysfunctions in autophagy, a cellular process essential for the removal of damaged organelles and proteins, disrupt cardiomyocyte homeostasis and lead to the accumulation of toxic cellular debris [31, 32]. Impaired mitochondrial maintenance reduces ATP production, increases oxidative stress, and heightens the heart’s vulnerability to energy deficits [33–37]. DNA repair mechanisms protect cardiomyocytes from genotoxic stress and ensuring genomic stability, with deficiencies potentially leading to apoptosis or senescence [38, 39]. Furthermore, disruptions in lipid metabolism [40–42], glycogen storage [43–45], and cellular signaling pathways[29, 46, 47] can drive hypertrophic and dilated cardiomyopathies by altering energy homeostasis and metabolic efficiency. The potential roles of these 46 genes in non-ischemic heart failure, as previously reported, are referenced in Supplementary Table 2.

The major limitation of our study is the small size owing to the fact that we collected samples and data from only a single institution and were constrained by the timing of the introduction of antibodies, vaccines and other treatment strategies. We also acknowledge that focusing solely on hospitalized patients may introduce selection bias, as this group represents a subset of the COVID-19 population with more severe disease manifestations. Follow-up studies are warranted to identify whether the differences in genetic variants seen in this first study of COVID-19 patients and prior studies in non-infectious DCM are attributable to differences in the genetic landscape or to fundamental biological differences in genomic variants across different ancestral groups. Tools like WES are indeed critical for studying rare variants, particularly in addressing minority populations and health disparities. This work provides a starting point for those studies, which may present a new perspective of precision medicine in both cardiology and infectious diseases. Also, given the study’s limited sample size, it is important to acknowledge its lack of statistical power to identify a specific gene as a novel contributor to viral heart injury. It did not include a comparable hospitalized control population without COVID. Additionally, the patients exhibited heterozygous phenotypes. Functional cardiac data, such as ejection fraction (EF) measured by transthoracic echocardiography (TTE), was not available for analysis, rendering comparison of cardiac function between subjects with and without potentially pathogenic variants more difficult. We envision that further research on available samples from existing SARS-CoV-2 studies may be able to address some of these limitations and confirm our findings. Expanding analyses to larger and more diverse cohorts will enhance the generalizability of our findings, helping to confirm they are not limited to specific populations. However, the success of vaccines and other treatment strategies necessitates larger sample sizes to effectively study cardiac injury in COVID-19 patients.

## Supplementary Information

Below is the link to the electronic supplementary material.

Supplementary file1 (DOCX 797 KB)

Supplementary file2 (XLSX 1880 KB)

Supplementary file3 (XLSX 22 KB)

Supplementary file4 (XLSX 926 KB)

## Data Availability

The data that support the findings of this study are available on request from the corresponding authors. The data are not publicly available due to privacy or ethical restrictions.
