# Supplementary material for: Rare variants in cardiomyopathy genes predispose to cardiac injury in severe COVID-19 patients of African or Hispanic ancestry
Source: J Mol Med (Berl). 2024 Dec 27;103(2):175–85. doi: 10.1007/s00109-024-02510-z (PMC11799050; doi:10.1007/s00109-024-02510-z)
Supplement: Supplementary file 1 — Supplementary file1 (DOCX 797 KB) [file 109_2024_2510_MOESM1_ESM.docx]

**Rare variants in cardiomyopathy genes predispose to cardiac injury in severe COVID-19 patients of African or Hispanic ancestry**

Hui-Qi Qu^1†^, Matthew S. Delfiner^2†^, Chethan Gangireddy^2^, Anjali Vaidya^2^, Kenny Nguyen^1^, Isaac R. Whitman^2^, JuFang Wang^2^, Jianliang Song^2^, Michael R. Bristow^3^, Charles F. McTiernan^4^, Glenn S. Gerhard^5^, Hakon Hakonarson^1,6,7*^, Arthur M. Feldman ^2*^

**Supplementary Information**

This supplementary information provides additional details on the methodologies, results, and discussions from our study that could not be included in the main text due to space limitations.

**Supplementary Methods:** **Protein extraction and western blot analysis for MYBPC3 and CDH23**

The left ventricular myocardium samples of failing and non-failing human heart (n=6 for each group) were obtained from transplant recipients whose diseased hearts were removed at the time of transplant surgery or from the normal hearts of organ donors whose hearts could not be used for transplant due to a lack of matching recipients but whose other organs were being harvested for transplant as described in detail previously. The samples were randomly selected from the heart tissue bank described in our previous work^1^, and included primarily individuals identified as European Americans and AA. Apart from disease status (failing vs. non-failing), clinical characteristics of these samples were blinded to the investigators to maintain ethical standards and ensure confidentiality. The hearts were flash-frozen in liquid nitrogen and stored at -80^o^ C in the cardiomyopathy biorepositories at the University of Pittsburgh School of Medicine and the University of Colorado School of Medicine. Consent was obtained from all tissue donors and the protocols were approved by the IRBs of the University of Pittsburgh and the University of Colorado. At the time of use, samples were homogenized in protein lysate (20 μl) mixed with a reducing agent (Thermo Fisher) and proteins were separated using a BoltTM 4-12% Bis-Tris Gel (Invitrogen) and transferred to nitrocellulose membranes using a wet transfer technique. Membranes were quickly washed and blocked with Licor Odyssey blocking buffer (LiCor) for 1 hour at room temperature before incubation with primary antibodies overnight at 4oC. The membranes were then washed with 1X PBS-T (0.1% Tween-20) and incubated with the secondary antibody for 2 hours at room temperature. The resulting images were captured and analyzed with a Licor imaging system.

**Supplementary Results:** **The role of *MYBPC3* and *CDH23* in the heart**

Genetic variants in *MYBC3* have been shown to be one of the two commonest genetic causes of familial hypertrophic cardiomyopathy ^2,3^; however, patients with genetic variants in *MYBC3* can also present with significant cardiac dysfunction and signs and symptoms of advanced heart failure due to severe diastolic dysfunction.^4,5^ While genetic variants in cadherin-23 (*CDH23*) have been primarily associated with both syndromic and non-syndromic hearing loss as well as the development of Usher Syndrome^6^, it is noteworthy that a novel variant in N-cadherin was recently associated with dilated cardiomyopathy^7^, whereas absence of *CDH23* or the presence of its excretory isoforms can loosen cell-cell adhesions that are required for optimal heart function.^8^ We therefore decided to ascertain levels of **MYBC3** and *CDH23* protein in the hearts of patients with end-stage heart failure to see if we could identify hearts in which either the protein levels of *MYBC3* or *CDH23* were altered independent of the presence of a SARS-CoV-19 infection. Using tissue from human hearts with non-ischemic dilated cardiomyopathy obtained at the time of heart transplant and samples from non-failing control hearts obtained from organ donors whose hearts could not be used for transplant due to an absence of appropriate recipients, we found no differences in the levels of *MYBC3* between failing and non-failing hearts. By contrast, *CDH23* protein levels were significantly and substantially reduced in every sample that we analyzed (Supplementary Fig. 1), suggesting for the first time that *CDH23* may play an important, albeit unrecognized role, in the genesis of dilated cardiomyopathy independent of genotype. We must acknowledge that these two genes were highlighted by our genetic study; however, we were unable to focus more comprehensively on these targets within the scope of this research. This finding from the western blot analysis is currently limited by the lack of detailed clinical characteristics for the samples analyzed, as clinical information beyond disease status (failing vs. non-failing) was blinded to the investigators. This limitation underscores the need for future studies with comprehensive clinical data to validate and better contextualize these results.

**Supplementary Discussion**

**Genetic insights into myocarditis**

Myocarditis remains an enigmatic disease and there has been little data available to suggest the cause of the disease, thereby obviating any possibility of either predicting those that might develop myocarditis or instituting preventive therapy. Belkaya et al were one of the first to evaluate the hypothesis that human genetic factors could underlie autosomal recessive cardiomyopathy.^9^ They performed WES of 42 unelated children with acute myocarditis and found that seven of 42 patients (16.7%) carried rare biallelic (homozygous or compound heterozygous) nonsynonymous or splice-site variations in 6 cardiomyopathy-associated genes (*BAG3, DSP, PKP2, RYR2, SCN5A or TNNI3*), demonstrating that rare homozygous or compound heterozygous variants in a subset of cardiomyopathy genes are disease causing in pediatric patients.

**Comparing myocarditis to DCM and COVID-19**

The aggregate data closely reflect the genotypic profile of cardiac diseases that are associated with DCM but do not have myocarditis. For example, amongst the 46 cardiomyopathy genes identified in this study are genetic variants that have been seen in other myopathies including *ACADS, COL7A1, CPT2, DSG2, LMNA, LAMB3, LDB3, SCN5A, TCAP, and TFR2*.^9-11^ Interestingly, while titin variants were present in this population, they were not at the level as seen in most studies of DCM. By contrast, we identified none of the genes previously identified in a GWAS of severe COVID-19 with respiratory failure that identified a 3p21.31 gene cluster that included potential involvement of the ABO blood-group system (*SLC6A20, LZTFL1, CCR9, FYCO1, CXCR6, XCR1*)^12^.

Our results also are not consistent with the results of a pan-ancestry exome-wide analyses of COVID-19 outcomes that was conducted in 586,157 individuals that did not identify any clear associations with rare variants either exome wide or when specifically focusing on selective interferon pathway genes (n=13), genes located in various susceptibility loci (n=281) or genes of immunologic and/or therapeutic (n=32) relevance.^13^ By contrast, our study focused on rare variants and uncovered multiple genetic variants that were associated with cardiac dysfunction in patients with severe COVID-19 disease. Further, our study focused almost exclusively on patient populations that have been excluded from most large exome-wide and genome-wide association analysis, individuals of African and Hispanic ancestry and thus comparator studies are lacking.

**Key scientific and clinical implications**

This study provides several pieces of information that are important both scientifically and clinically. First, we show for the first time that rare genetic variants can alter the molecular landscape of hearts harboring infections with SARS-CoV-2 and that while many of the same genes that are involved in the cardiomyopathy phenotype are seen with COVID-19, the proportions of specific variants are quite different. Second, this study represents one of the largest proportions of subjects in a heart failure-related genetic study who are of African ancestry. And third, we identify new potential therapeutic targets with levels that are significantly diminished in the failing human heart, such as the substantial decrease in cadherin-23.

**
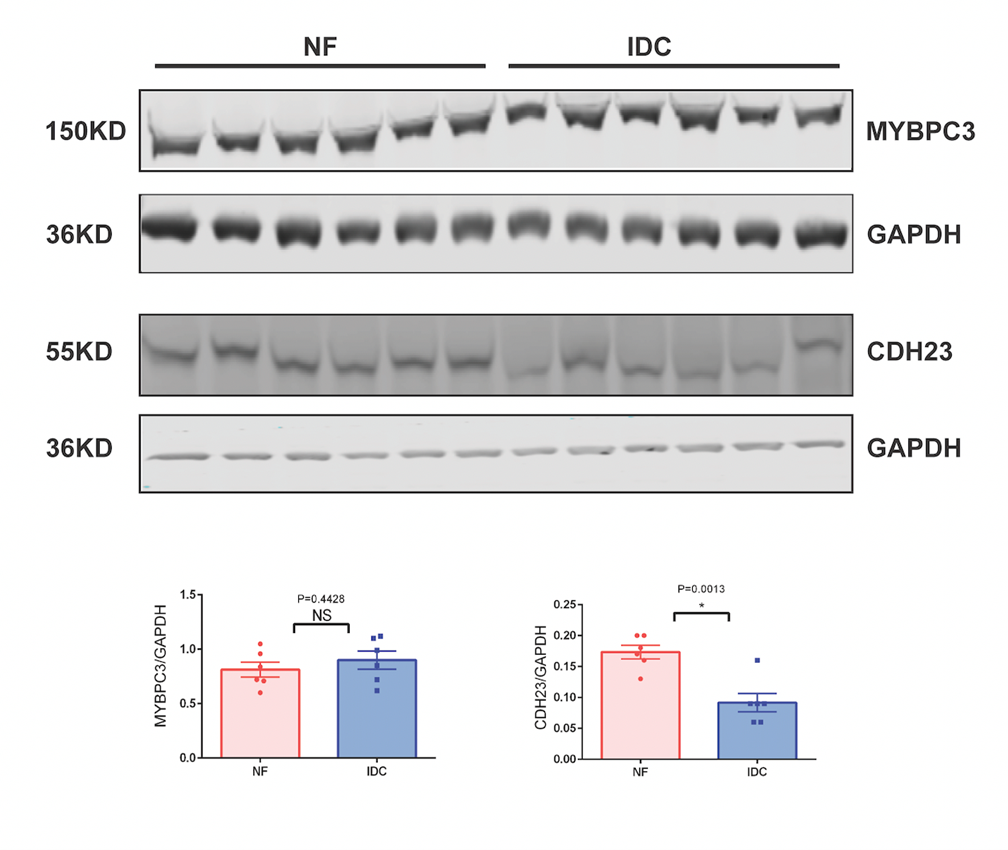
**

**Supplementary Figure 1.** Western blot analysis of levels of Cadherin 23 (CDH23) and myosin binding protein C (MYBPC3) in failing (IDC) and non-failing (NF) hearts with preserved ventricular function.

**Supplementary Table 1** HPO gene sets enriched with statistical significance.

**Supplementary Table 2** Evidence from the literature for the cardiomyopathy genes with HD variants.

**Supplementary Data 1** 26,661 variants of interest (VOI).

**Supplementary Data 2** 263 highly deleterious (HD) variants.

**Supplementary data 3** Mutation burden analysis of variants of interest.

**Supplementary Table 1** HPO gene sets enriched with statistical significance.

| **Gene Set** | **Description** | **Size** | **Expect** | **Ratio** | **Number of Genes with HD variants** | **P Value** | **FDR** |
| --- | --- | --- | --- | --- | --- | --- | --- |
| HP:0001638 | Cardiomyopathy | 379 | 19.726 | 2.3319 | 46 | 1.51E-08 | 7.08E-05 |
| HP:0001637 | Abnormal myocardium morphology | 401 | 20.871 | 2.2519 | 47 | 3.16E-08 | 7.38E-05 |
| HP:0001789 | Hydrops fetalis | 49 | 2.5504 | 5.4894 | 14 | 9.19E-08 | 0.000114 |
| HP:0001939 | Abnormality of metabolism/homeostasis | 1819 | 94.676 | 1.3837 | 131 | 9.79E-08 | 0.000114 |
| HP:0011277 | Abnormality of the urinary system physiology | 730 | 37.995 | 1.7897 | 68 | 1.76E-07 | 0.000165 |
| HP:0001626 | Abnormality of the cardiovascular system | 1774 | 92.334 | 1.343 | 124 | 3.13E-06 | 0.002436 |
| HP:0003110 | Abnormality of urine homeostasis | 455 | 23.682 | 1.9424 | 46 | 3.81E-06 | 0.002542 |
| HP:0004354 | Abnormality of carboxylic acid metabolism | 218 | 11.347 | 2.4677 | 28 | 5.14E-06 | 0.003003 |
| HP:0001662 | Bradycardia | 43 | 2.2381 | 4.9149 | 11 | 7.65E-06 | 0.003977 |
| HP:0012072 | Aciduria | 164 | 8.5359 | 2.6945 | 23 | 8.94E-06 | 0.004179 |

**Supplementary Table 2 Evidence from the literature for the cardiomyopathy genes with HD variants**

| **Gene Symbol** | **Variant** | **Count_Afr** | **Count_NonAfr** | **subtotal** | **ExAC_AFR** | **ExAC_Freq (all)** | **P_AFR** | **P_NFE** | **Fold change^#^** | **adj.P.Val^#^** | **Gene Name** | **Gene Notes** |
| --- | --- | --- | --- | --- | --- | --- | --- | --- | --- | --- | --- | --- |
| *ACADS* | A385S | 0 | 2 | 2 | 0 | 4.33E-05 | 4.79E-04 | 4.95E-08 | 1.076 | 0.300 | acyl-CoA dehydrogenase short chain | Hypertrophic and dilated cardiomyopathies in patients with *ACADS* gene susceptibility variants^14^ |
| *AGK* | Y224X | 0 | 1 | 1 | 0 | 2.49E-05 | 5.76E-02 | 2.38E-03 | 1.152 | 0.013* | acylglycerol kinase | Mutations cause cardiomyopathy and cataracts (Sengers syndrome)^15^ |
| *AGL* | Q86X | 1 | 0 | 1 | 0.0003 | 2.48E-05 | 5.63E-05 | 4.78E-02 | 1.293 | 0.122 | amylo-alpha-1, 6-glucosidase, 4-alpha-glucanotransferase | Mutations cause glycogen storage disease type IIIa with hypertrophic cardiomyopathy^16^ |
| *ANO5* | N51S | 0 | 1 | 1 | 0.0007 | 0.0019 | 8.88E-08 | 1.84E-04 |  |  | anoctamin 5 | Dilated cardiomyopathy in patients with mutations in anoctamin 5^17^ |
|  | G230V | 0 | 1 | 1 | 0.0002 | 0.0011 |  |  |  |  |  |  |
| *BCS1L* | R56X | 0 | 1 | 1 | 9.61E-05 | 0.0002 | 1.57E-01 | 1.74E-02 | 1.000 | 0.996 | BCS1 homolog, ubiquinol-cytochrome c reductase complex chaperone | Non-obstructive hypertrophic cardiomyopathy observed in 2 patients with *BCS1L* variants^18^ |
| *BRCA1* | A1661V | 2 | 0 | 2 | 0.0004 | 3.32E-05 | 5.21E-07 | 2.01E-05 | 1.054 | 0.223 | BRCA1, DNA repair associated | BRCA1 shields cardiomyocytes from DNA damage, apoptosis and heart dysfunction^19^ |
| *BRIP1* | P47A | 0 | 1 | 1 | 0 | 0.0002 | 3.52E-03 | 2.79E-02 | 1.046 | 0.305 | BRCA1 interacting protein C-terminal helicase 1 | Binding of BRIP1 is required for the function of BRCA1^20^ |
| *CAV3* | C72W | 1 | 1 | 2 | 0.0006 | 0.0011 | 7.69E-02 | 1.86E-01 | 1.103 | 0.082 | caveolin 3 | Mutation causes familiar hypertrophic cardiomyopathy^21^ |
| *CDH23* | R1060W | 1 | 1 | 2 | 0.0002 | 0.0004 | 4.44E-08 | 9.46E-08 |  |  | cadherin related 23 | Genetic association with cardiac troponin T measurement^22^ |
| *COL7A1* | R2580C | 0 | 1 | 1 | 0 | 3.96E-05 | 8.72E-06 | 2.40E-02 | 0.970 | 0.721 | collagen type VII alpha 1 chain | Dilated cardiomyopathy in dystrophic epidermolysis bullosa with *COL7A1* mutation^23^ |
| *CPT2* | S113L | 0 | 2 | 2 | 0.0002 | 0.0013 | 4.44E-03 | 2.70E-02 | 1.185 | 0.012* | carnitine palmitoyltransferase 2 | Cardiomyopathy caused by *CPT2* mutation^24^ |
| *DSG2* | G812S | 0 | 1 | 1 | 0 | 1.66E-05 | 3.11E-03 | 2.30E-03 | 1.279 | 2.76E-03* | desmoglein 2 | Cardiomyopathy caused by *DSG2* mutation^25^ |
| *ELAC2* | R741H | 0 | 1 | 1 | 0.0004 | 0.0005 | 4.71E-02 | 1.65E-02 | 1.034 | 0.438 | elaC ribonuclease Z 2 | Hypertrophic cardiomyopathy caused by *ELAC2* mutation^26^ |
| *FAH* | G337S | 1 | 0 | 1 | 0.0003 | 0.0001 | 3.79E-02 | 2.42E-04 | 0.995 | 0.976 | fumarylacetoacetate hydrolase | Hypertrophic cardiomyopathy caused by *FAH* mutation^27^ |
| *FANCA* | R853X | 1 | 0 | 1 | 9.69E-05 | 8.26E-06 | 5.04E-05 | 9.36E-11 | 0.969 | 0.521 | FA complementation group A | Dilated cardiomyopathy in Fanconi anemia^28^ |
| *FBXL4* | Q597P | 0 | 1 | 1 | 0 | 1.65E-05 | 5.93E-02 | 2.88E-02 | 0.985 | 0.775 | F-box and leucine rich repeat protein 4 | Hypertrophic cardiomyopathy caused by *FBXL4* mutation^29^ |
| *FIG4* | I41T | 0 | 1 | 1 | 9.91E-05 | 0.001 | 1.39E-02 | 8.68E-02 | 1.108 | 0.065 | FIG4 phosphoinositide 5-phosphatase | Cardiomyopathy in defective autophagy^30^ |
| *GBE1* | Y329S | 0 | 1 | 1 | 0 | 0.0003 | 1.12E-03 | 3.50E-01 | 0.759 | 7.39E-04* | 1,4-alpha-glucan branching enzyme 1 | Dilated cardiomyopathy in patients with *GBE1* mutation^31^ |
|  | c.691+2T>C | 1 | 0 | 1 | 0 | 0.0011 |  |  |  |  |  |  |
| *GNPTAB* | A592T | 1 | 0 | 1 | 0 | 0.0001 | 1.44E-06 | 4.11E-04 | 1.134 | 0.178 | N-acetylglucosamine-1-phosphate transferase subunits alpha and beta | Dilated cardiomyopathy in patients with *GNPTAB* mutation^32^ |
| *GYG1* | D102H | 1 | 0 | 1 | 0.0002 | 0.0009 | 1.67E-01 | 6.07E-01 | 0.776 | 5.30E-04* | glycogenin 1 | Cardiomyopathy caused by *GYG1* mutation^33^ |
| *HADHA* | E510Q | 1 | 0 | 1 | 0.0004 | 0.0012 | 5.69E-04 | 2.58E-03 | 0.826 | 0.299 | hydroxyacyl-CoA dehydrogenase trifunctional multienzyme complex subunit alpha | Cardiomyopathy caused by *HADHA* mutation^34^ |
| *IDUA* | R628X | 0 | 1 | 1 | 0 | 3.35E-05 | 2.81E-04 | 1.96E-04 | 1.071 | 0.107 | iduronidase, alpha-L- | Cardiomyopathy in Hurler syndrome with IDUA deficiency^35^ |
|  | Q70X | 0 | 1 | 1 | 0.0001 | 0.0007 |  |  |  |  |  |  |
| *ITGA7* | R143X | 1 | 0 | 1 | 0.0002 | 4.12E-05 | 3.64E-02 | 8.55E-06 | 0.800 | 0.093 | integrin subunit alpha 7 | Cardiac dysfunction by *ITGA7* mutation^36^ |
| *LAMB3* | R635X | 0 | 1 | 1 | 0.0002 | 0.0008 | 7.68E-03 | 1.42E-01 | 0.905 | 0.018* | laminin subunit beta 3 | Cardiomyopathy in hereditary epidermolysis bullosa^37^ |
| *LDB3* | S189L | 0 | 1 | 1 | 0 | 0.0006 | 8.53E-02 | 3.45E-03 | 1.538 | 4.48E-03* | LIM domain binding 3 | Cardiomyopathy caused by *LDB3* mutation^38^ |
| *LMNA* | S543L | 0 | 1 | 1 | 0 | 0.0001 | 8.28E-02 | 1.07E-01 | 1.053 | 0.555 | lamin A/C | LMNA cardiomyopathy^39^ |
| *MYBPC3* | Q998E | 0 | 5 | 5 | 0.0004 | 0.0052 | 5.44E-10 | 1.05E-07 | 1.139 | 0.287 | myosin binding protein C, cardiac | Pathogenic variants in patients with hypertrophic cardiomyopathy^40^ |
|  | E542Q | 1 | 0 | 1 | 0.0001 | 2.49E-05 |  |  |  |  |  |  |
| *MYLK2* | A87V | 0 | 1 | 1 | 0.0003 | 0.0001 | 4.06E-01 | 6.39E-01 |  |  | myosin light chain kinase 2 | Cardiomyopathy caused by *MYLK2* mutation^41^ |
| *NAGLU* | R626X | 1 | 0 | 1 | 0.0002 | 2.74E-05 | 1.35E-03 | 1.45E-05 | 0.934 | 0.343 | N-acetyl-alpha-glucosaminidase | Cardiac disease in Sanfilippo syndrome B by *NAGLU* mutation^42^ |
| *OPA1* | I346M | 0 | 1 | 1 | 0.0004 | 0.0006 | 3.11E-05 | 1.44E-02 | 1.275 | 2.32E-03* | OPA1, mitochondrial dynamin like GTPase | Cardiomyopathy caused by *OPA1* mutation^43^ |
| *PEX1* | G786D | 0 | 1 | 1 | 9.71E-05 | 0.0003 | 4.96E-05 | 1.24E-06 | 1.072 | 0.035* | peroxisomal biogenesis factor 1 | Hypertrophic cardiomyopathy in Zellweger syndrome^44^ |
| *PEX10* | R311Q | 1 | 0 | 1 | 0.0005 | 3.77E-05 | 3.42E-02 | 1.01E-07 | 1.129 | 0.044* | peroxisomal biogenesis factor 10 | Hypertrophic cardiomyopathy in Zellweger syndrome^44^ |
| *PIGT* | R346W | 1 | 0 | 1 | 0 | 1.65E-05 | 1.28E-03 | 6.29E-03 | 1.192 | 7.73E-03* | phosphatidylinositol glycan anchor biosynthesis class T | Restrictive cardiomyopathy in an intellectual disability syndrome by *PIGT* mutations^45^ |
| *POLG* | G737R | 0 | 1 | 1 | 0.0003 | 0.0007 | 1.35E-05 | 2.49E-04 | 0.926 | 0.244 | DNA polymerase gamma, catalytic subunit | Cardiomyopathy caused by *POLG* mutation^46^ |
| *PPA2* | P62L | 0 | 1 | 1 | 9.89E-05 | 0.0002 | 1.17E-01 | 2.09E-01 | 1.039 | 0.688 | pyrophosphatase (inorganic) 2 | Cardiomyopathy caused by *PPA2* mutation^47^ |
| *SCN5A* | R1808H | 2 | 0 | 2 | 0.0001 | 3.31E-05 | 1.79E-05 | 5.30E-06 | 1.024 | 0.873 | sodium voltage-gated channel alpha subunit 5 | Dilated cardiomyopathy by *SCN5A* mutation^48^ |
| *SLC25A20* | c.198+2T>C | 1 | 0 | 1 | 0.0006 | 4.94E-05 | 3.47E-01 | 9.68E-03 | 1.174 | 0.022* | solute carrier family 25 member 20 | Carnitine acyl-carnitine translocase deficiency with cardiomyopathy by *SLC25A20* mutation^49^ |
| *SLC2A10* | Y139X | 0 | 1 | 1 | 0 | 8.24E-06 | 5.90E-02 | 1.93E-02 | 1.267 | 7.34E-06* | solute carrier family 2 member 10 | Heart failure and myocarditis in arterial tortuosity syndrome by *SLC2A10* mutation^50^ |
| *SURF1* | R83Q | 1 | 0 | 1 | 0 | 1.80E-05 | 6.73E-02 | 4.89E-02 | 1.150 | 0.072 | SURF1, cytochrome c oxidase assembly factor | Cardiomyopathy by SURF1 deficiency^51^ |
|  | R121X | 1 | 0 | 1 | 9.63E-05 | 0.0002 |  |  |  |  |  |  |
| *TCAP* | R70Q | 0 | 2 | 2 | 0 | 2.09E-05 | 1.11E-03 | 1.27E-06 | 1.176 | 0.129 | titin-cap | Hypertrophic cardiomyopathy and dilated cardiomyopathy by *TCAP* mutations^52^ |
| *TFR2* | R297H | 1 | 0 | 1 | 9.83E-05 | 0.0007 | 5.76E-03 | 2.87E-01 | 0.962 | 0.499 | transferrin receptor 2 | Cardiomyopathy in type 3 hemochromatosis by *TFR2* mutation^53^ |
| *TRIP4* | R79X | 0 | 1 | 1 | 0 | 2.47E-05 | 1.00E-02 | 3.22E-03 | 1.092 | 0.260 | thyroid hormone receptor interactor 4 | Cardiomyopathy caused by *TRIP4* mutation^54^ |
| *TTN* | R16487X | 0 | 1 | 1 | 0 | 8.34E-06 | 1.02E-39 | 1.12E-05 | 0.983 | 0.900 | titin | Cardiomyopathy caused by *TTN* mutation^55^ |
| *TTR* | F64L | 2 | 0 | 2 | 0.0006 | 5.77E-05 | 7.66E-02 | 2.78E-04 | 0.934 | 0.234 | transthyretin | TTR cardiomyopathy^56^ |
| *VPS13A* | R1258X | 0 | 2 | 2 | 0 | 8.24E-06 | 5.53E-06 | 6.70E-04 | 1.136 | 0.144 | vacuolar protein sorting 13 homolog A | Cardiomyopathy in VPS13A Disease^57^ |
| *WFS1* | G674R | 0 | 1 | 1 | 0 | 0.0003 | 4.64E-06 | 6.32E-02 | 1.382 | 6.19E-04* | wolframin ER transmembrane glycoprotein | Wolframin expression in heart and heart diseases in Wolfram syndrome 1^58^ |

P_AFR: burden test of rare coding variants compared to the population frequencies in the ExAC African population; P_NFE: burden test of rare coding variants compared to the population frequencies in the ExAC Non-Finish European (NFE) population.

^#^Differential expression in idiopathic cardiomyopathy with adjusted P value<0.05, according to the large sample study by Dr. Thomas Cappola at the Univeristy of Pennsylvania School of Medicine^59^, including 16 controls and 86 idiopathic cardiomyopathy.

References

1 Bedi, M. S. *et al.* Myocardial Fas and cytokine expression in end‐stage heart failure: impact of LVAD support. *Clinical and Translational Science* **1**, 245-248 (2008).

2 Seidman, J. G. & Seidman, C. The genetic basis for cardiomyopathy: from mutation identification to mechanistic paradigms. *Cell* **104**, 557-567, doi:10.1016/s0092-8674(01)00242-2 (2001).

3 Erdmann, J. *et al.* Mutation spectrum in a large cohort of unrelated consecutive patients with hypertrophic cardiomyopathy. *Clin Genet* **64**, 339-349, doi:10.1034/j.1399-0004.2003.00151.x (2003).

4 Bahrudin, U. *et al.* Ubiquitin-proteasome system impairment caused by a missense cardiac myosin-binding protein C mutation and associated with cardiac dysfunction in hypertrophic cardiomyopathy. *J Mol Biol* **384**, 896-907, doi:10.1016/j.jmb.2008.09.070 (2008).

5 Naito, S. *et al.* End-stage Hypertrophic Cardiomyopathy with Advanced Heart Failure in Patients Carrying MYH7 R453 Variants: A Case Series. *Intern Med*, doi:10.2169/internalmedicine.1497-22 (2023).

6 Kang, B. *et al.* Identification of four novel variants in the CDH23 gene from four affected families with hearing loss. *Front Genet* **13**, 1027396, doi:10.3389/fgene.2022.1027396 (2022).

7 Chen, Y. *et al.* Identification of a novel variant in N-cadherin associated with dilated cardiomyopathy. *Front Med (Lausanne)* **9**, 944950, doi:10.3389/fmed.2022.944950 (2022).

8 Li, Y. *et al.* The N-cadherin interactome in primary cardiomyocytes as defined using quantitative proximity proteomics. *J Cell Sci* **132**, doi:10.1242/jcs.221606 (2019).

9 Belkaya, S. *et al.* Autosomal Recessive Cardiomyopathy Presenting as Acute Myocarditis. *J Am Coll Cardiol* **69**, 1653-1665, doi:10.1016/j.jacc.2017.01.043 (2017).

10 Ware, J. S. *et al.* Shared Genetic Predisposition in Peripartum and Dilated Cardiomyopathies. *N Engl J Med* **374**, 233-241, doi:10.1056/NEJMoa1505517 (2016).

11 Mazzarotto, F. *et al.* Reevaluating the Genetic Contribution of Monogenic Dilated Cardiomyopathy. *Circulation* **141**, 387-398, doi:10.1161/CIRCULATIONAHA.119.037661 (2020).

12 Group, S. C.-G. Genomewide association study of severe Covid-19 with respiratory failure. *New England Journal of Medicine* **383**, 1522-1534 (2020).

13 Kosmicki, J. A. *et al.* Pan-ancestry exome-wide association analyses of COVID-19 outcomes in 586,157 individuals. *Am J Hum Genet* **108**, 1350-1355, doi:10.1016/j.ajhg.2021.05.017 (2021).

14 Dessein, A.-F. *et al.* Fluxomic evidence for impaired contribution of short-chain acyl-CoA dehydrogenase to mitochondrial palmitate β-oxidation in symptomatic patients with ACADS gene susceptibility variants. *Clinica Chimica Acta* **471**, 101-106, doi:<https://doi.org/10.1016/j.cca.2017.05.026> (2017).

15 Siriwardena, K. *et al.* Mitochondrial citrate synthase crystals: novel finding in Sengers syndrome caused by acylglycerol kinase (AGK) mutations. *Molecular genetics and metabolism* **108**, 40-50 (2013).

16 Lucchiari, S. *et al.* Clinical and genetic variability of glycogen storage disease type IIIa: seven novel AGL gene mutations in the Mediterranean area. *American journal of medical genetics* **109**, 183-190 (2002).

17 Wahbi, K. *et al.* Dilated cardiomyopathy in patients with mutations in anoctamin 5. *International journal of cardiology* **168**, 76-79 (2013).

18 Al-Owain, M. *et al.* Clinical and biochemical features associated with BCS1L mutation. *Journal of inherited metabolic disease* **36**, 813-820 (2013).

19 Shukla, P. C. *et al.* BRCA1 is an essential regulator of heart function and survival following myocardial infarction. *Nature Communications* **2**, 593, doi:10.1038/ncomms1601 (2011).

20 Cantor, S. B. *et al.* BACH1, a novel helicase-like protein, interacts directly with BRCA1 and contributes to its DNA repair function. *Cell* **105**, 149-160, doi:10.1016/s0092-8674(01)00304-x (2001).

21 Hayashi, T. *et al.* Identification and functional analysis of a caveolin-3 mutation associated with familial hypertrophic cardiomyopathy. *Biochemical and biophysical research communications* **313**, 178-184, doi:10.1016/j.bbrc.2003.11.101 (2004).

22 Yang, Y. *et al.* Identification of Functional Genetic Determinants of Cardiac Troponin T and I in a Multiethnic Population and Causal Associations With Atrial Fibrillation. *Circulation. Genomic and precision medicine* **14**, e003460, doi:10.1161/circgen.121.003460 (2021).

23 Sidwell, R. U., Yates, R. & Atherton, D. Dilated cardiomyopathy in dystrophic epidermolysis bullosa. *Archives of Disease in Childhood* **83**, 59-63, doi:10.1136/adc.83.1.59 (2000).

24 Longo, N., Amat di San Filippo, C. & Pasquali, M. in *American Journal of Medical Genetics Part C: Seminars in Medical Genetics.* 77-85 (Wiley Online Library).

25 Awad, M. M. *et al.* DSG2 mutations contribute to arrhythmogenic right ventricular dysplasia/cardiomyopathy. *The American Journal of Human Genetics* **79**, 136-142 (2006).

26 Haack, T. B. *et al.* ELAC2 mutations cause a mitochondrial RNA processing defect associated with hypertrophic cardiomyopathy. *The American Journal of Human Genetics* **93**, 211-223 (2013).

27 Mohamed, S. *et al.* Tyrosinemia type 1: a rare and forgotten cause of reversible hypertrophic cardiomyopathy in infancy. *BMC Research Notes* **6**, 1-4 (2013).

28 Park, H.-Y. Hereditary dilated cardiomyopathy: recent advances in genetic diagnostics. *Korean Circulation Journal* **47**, 291-298 (2017).

29 Sabouny, R. *et al.* Characterization of the C584R variant in the mtDNA depletion syndrome gene FBXL4, reveals a novel role for FBXL4 as a regulator of mitochondrial fusion. *Biochimica et Biophysica Acta (BBA)-Molecular Basis of Disease* **1865**, 165536 (2019).

30 Deneubourg, C. *et al.* The spectrum of neurodevelopmental, neuromuscular and neurodegenerative disorders due to defective autophagy. *Autophagy* **18**, 496-517 (2022).

31 Fernandez, C. *et al.* Non‐lethal neonatal neuromuscular variant of glycogenosis type IV with novel GBE1 mutations. *Muscle & Nerve: Official Journal of the American Association of Electrodiagnostic Medicine* **41**, 269-271 (2010).

32 Carboni, E. *et al.* Dilated cardiomyopathy in mucolipidosis type 2. *Journal of Biological Regulators and Homeostatic Agents* **34**, 71-77. SPECIAL ISSUE: FOCUS ON PEDIATRIC CARDIOLOG (2020).

33 Hedberg‐Oldfors, C. *et al.* Cardiomyopathy as presenting sign of glycogenin‐1 deficiency—report of three cases and review of the literature. *Journal of Inherited Metabolic Disease: Official Journal of the Society for the Study of Inborn Errors of Metabolism* **40**, 139-149 (2017).

34 Ojala, T. *et al.* Fetal left ventricular noncompaction cardiomyopathy and fatal outcome due to complete deficiency of mitochondrial trifunctional protein. *European journal of pediatrics* **174**, 1689-1692 (2015).

35 Braunlin, E. *et al.* Cardiac Functional and Histopathologic Findings in Humans and Mice with Mucopolysaccharidosis Type I: Implications for Assessment of Therapeutic Interventions in Hurler Syndrome. *Pediatric Research* **59**, 27-32, doi:10.1203/01.pdr.0000190579.24054.39 (2006).

36 Bugiardini, E. *et al.* Integrin α7 Mutations Are Associated With Adult‐Onset Cardiac Dysfunction in Humans and Mice. *Journal of the American Heart Association* **11**, e026494 (2022).

37 Will, L. M., Reichrath, J. & Vogt, T. Epidermolysis bullosa dystrophica pretibialis – Clinical snapshot and management of a rare orphan disease. *JDDG: Journal der Deutschen Dermatologischen Gesellschaft* **19**, 983-986, doi:<https://doi.org/10.1111/ddg.14446> (2021).

38 Koopmann, T. T. *et al.* Biallelic loss of LDB3 leads to a lethal pediatric dilated cardiomyopathy. *European Journal of Human Genetics* **31**, 97-104 (2023).

39 Lu, J. T., Muchir, A., Nagy, P. L. & Worman, H. J. LMNA cardiomyopathy: cell biology and genetics meet clinical medicine. *Disease models & mechanisms* **4**, 562-568 (2011).

40 Helms, A. S. *et al.* Spatial and functional distribution of MYBPC3 pathogenic variants and clinical outcomes in patients with hypertrophic cardiomyopathy. *Circulation: Genomic and Precision Medicine* **13**, 396-405 (2020).

41 Qin, X. *et al.* FLNC and MYLK2 gene mutations in a Chinese family with different phenotypes of cardiomyopathy. *International Heart Journal* **62**, 127-134 (2021).

42 Lavery, C., Hendriksz, C. J. & Jones, S. A. Mortality in patients with Sanfilippo syndrome. *Orphanet J Rare Dis* **12**, 168, doi:10.1186/s13023-017-0717-y (2017).

43 Chen, L. *et al.* OPA 1 Mutation and Late‐Onset Cardiomyopathy: Mitochondrial Dysfunction and mtDNA Instability. *Journal of the American Heart Association* **1**, e003012 (2012).

44 Kale, Y. *et al.* A case of Zellweger syndrome accompanied by hypertrophic cardiomyopathy. *Medical Science and Discovery* **3**, 242-244 (2016).

45 Kvarnung, M. *et al.* A novel intellectual disability syndrome caused by GPI anchor deficiency due to homozygous mutations in PIGT. *Journal of medical genetics* **50**, 521-528 (2013).

46 Spracklen, T. F. *et al.* Identification of a POLG Variant in a Family With Arrhythmogenic Cardiomyopathy and Left Ventricular Fibrosis. *Circulation: Genomic and precision medicine* **14**, e003138 (2021).

47 Vasilescu, C. *et al.* Genetic basis of severe childhood-onset cardiomyopathies. *Journal of the American College of Cardiology* **72**, 2324-2338 (2018).

48 McNair, W. P. *et al.* SCN5A mutation associated with dilated cardiomyopathy, conduction disorder, and arrhythmia. *Circulation* **110**, 2163-2167 (2004).

49 Iacobazzi, V. *et al.* Molecular and functional analysis of SLC25A20 mutations causing carnitine‐acylcarnitine translocase deficiency. *Human mutation* **24**, 312-320 (2004).

50 Callewaert, B. *et al.* Arterial tortuosity syndrome: clinical and molecular findings in 12 newly identified families. *Human mutation* **29**, 150-158 (2008).

51 Wedatilake, Y. *et al.* SURF1 deficiency: a multi-centre natural history study. *Orphanet J Rare Dis* **8**, 96, doi:10.1186/1750-1172-8-96 (2013).

52 Hayashi, T. *et al.* Tcap gene mutations in hypertrophic cardiomyopathy and dilated cardiomyopathy. *J Am Coll Cardiol* **44**, 2192-2201, doi:10.1016/j.jacc.2004.08.058 (2004).

53 Tang, S. *et al.* A Novel Mutation of Transferrin Receptor 2 in a Chinese Pedigree With Type 3 Hemochromatosis: A Case Report. *Front Genet* **13**, 836431, doi:10.3389/fgene.2022.836431 (2022).

54 Johannsen, J. *et al.* A Novel Nonsense Mutation in TRIP4 Gene Causes Severe Muscular Weakness with Respiratory Failure and Cardiomyopathy but without Skin, Joint, and/or Bone Abnormalities. *Neuropediatrics* **48**, PP07, doi:10.1055/s-0037-1602929 (2017).

55 Chauveau, C., Rowell, J. & Ferreiro, A. A rising titan: TTN review and mutation update. *Hum Mutat* **35**, 1046-1059, doi:10.1002/humu.22611 (2014).

56 Ruberg, F. L. & Berk, J. L. Transthyretin (TTR) cardiac amyloidosis. *Circulation* **126**, 1286-1300 (2012).

57 Peikert, K., Hermann, A. & Danek, A. XK-Associated McLeod Syndrome: Nonhematological Manifestations and Relation to VPS13A Disease. *Transfusion medicine and hemotherapy : offizielles Organ der Deutschen Gesellschaft fur Transfusionsmedizin und Immunhamatologie* **49**, 4-12, doi:10.1159/000521417 (2022).

58 Rigoli, L. & Di Bella, C. Wolfram syndrome 1 and Wolfram syndrome 2. *Current opinion in pediatrics* **24**, 512-517 (2012).

59 Hannenhalli, S. *et al.* Transcriptional genomics associates FOX transcription factors with human heart failure. *Circulation* **114**, 1269-1276, doi:10.1161/circulationaha.106.632430 (2006).
